# Supplementary material for: Anti-Obesity Effects of Ecklonia cava Extract in High-Fat Diet-Induced Obese Rats
Source: Antioxidants (Basel). 2022 Feb 3;11(2):310. doi: 10.3390/antiox11020310 (PMC8868354; doi:10.3390/antiox11020310)
Supplement: Supplementary file 1 [file antioxidants-11-00310-s001.zip › antioxidants-1582681-supplementary.pdf]

Anti-obesity effects of *Ecklonia cava* extract in high-fat diet-induced obese rats

(Supplementary Data)

Table S1. Base sequences of adipogenic and lipogenic primers with their PCR conditions.

Primers	Base Sequences	Polymerase Chain Reaction Conditions
PPAR-γ	F: CCA GAG TCT GCT GAT CTG CG<br>R: GCC ACC TCT TTG CTC TGA TC	Denaturation at 95 C for 45 s, annealing at 58 °C for 45 s, and synthesizing at 72 °C for 45 s with 35 cycles
FAS	F: GGCTCAGCATGGTCGCTT<br>R: CTCCCGCCAGCTGTCATT	Denaturation at 94 °C for 30 s, annealing at 60 °C for 45 s, and synthesizing at 72 °C for 30 s with 35 cycles
LPL	F: GATTTCTCTGTACGGCACAGTGG<br>R: TTTGTGGAAACCTCGGGC	Denaturation at 94 °C for 30 s, annealing at 58 °C for 45 s, and synthesizing at 72 °C for 30 s with 35 cycles
SREBP-1C	F: GTAGCGTCTGCACGCCCTA<br>R:CTTGGTTGTTGATGAGCTGGAG	Denaturation at 94 °C for 45 s, annealing at 58 °C for 45 s, and synthesizing at 72 °C for 45 s with 35 cycles
β -actin	F: ATGCTCCTGCTTGAGT A GT,<br>R: GAGGAAGAGGATGCGGCAGT	Denaturation at 95 °C for 45 s, annealing at 55 °C for 45 s, and synthesizing at 72 °C for 45 s with 30 cycles

*F, forward; R, reverse.

Table S2. The important bioactive compound in *E. cava* was identified by GCMS analysis.

	Compounds	RT	Area (%)	Functions	Chemical Formula	Ref.
**1**	Benzoyl bromide	27.080	10.84	Antibacterial, Antifungal, antioxidant, and anti-obesity activity.	C_7H_5BrO	[1] [2]
**2**	2-Propanol	27.764	3.23	Antibacterial properties and anti-prions activity with combinations.	C_3H_8O	PubChem
**3**	Benzene, 1,3-bis(1,1-dimethylethyl)-	36.529	2.95	It could be used as the base data for the effect of γ-irradiation on the medicinal herb.	$C_{14}H_{22}$	[3]
**4**	Docosane	37.478	2.34	The active ingredient in plant extract has antioxidant and anti-obesity and antibacterial activities.	$C_{22}H_{46}$	[4,5]
**5**	Dodecanal	53.494	3.14	Antibacterial activity and plant extracts which are recently reported for their significant anti-obesity, dodecanal was abundantly present.	$C_{12}H_{24}O$	[6-8]
**6**	Methyl salicylate	57.019	62.5	Analgesic, counter-irritant, and anti-inflammatory activities.	$C_8H_8O_3$	[8,9]
**7**	Tris(tert-butyldimethylsilyloxy)arsane	59.083	1.76	Antifungal, antibacterial, and antifungal. Abundantly present in the polyherbal extract which was reported for its anti-obesity effects.	$C_{18}H_{45}AsO_3Si_3$	[10,11]
	Arsenous acid	59.083	1.76	Anticancer activity	AsH_3O_3	[12]
**8**	Benzaldehyde, 4-propyl-	59.749	2.02	Antimicrobial activity	$C_{10}H_{12}O$	[13]
**9**	2,4-Di-tert-butylphenol	83.226	9.26	Antifungal, antioxidant, Anti-inflammatory activities.	$C_{14}H_{22}O$	[14,15]

RT—retention time.

Table S3. Effects of *Ecklonia cava* ethanol (70%) extract on major organs including liver, spleen, and kidney of rats fed with normal diet (NC), a high-fat diet (HFD), or HFD accompanied with treatment ECE at different doses of 125 mg (T1), 250 mg (T2), and 500 mg (T3) per kg B.W.

Groups	Liver wt. (gm/rat)	Spleen wt. (gm/rat)	Kidney wt. (gm/rat)
**NC**	12.67 ± 1.34	0.78 ± 0.14	1.60 ± 0.22*
**HFD**	17.82 ± 1.80^{+++}	0.97 ± 0.22	1.92 ± 0.22^{+}
**HCA (250 mg/kg)**	13.89 ± 2.5*	0.98 ± 0.26	1.82 ± 0.17
**EC (125 mg/kg)**	14.78 ± 2.97	1.0 ± 0.19	1.80 ± 0.33
**EC (250 mg/kg)**	13.84 ± 2.71*	0.82 ± 0.129	1.72 ± 0.15
**EC (500 mg/kg)**	12.76 ± 1.89***	0.79 ± 0.16	1.64 ± 0.04*

Data are presented as means ± SEM (n = 8 for each group). *$p < 0.05$, ** $p < 0.01$, *** $p < 0.001$ vs. HFD group, + $p < 0.05$, ++ $p < 0.01$, and +++ $p < 0.001$ vs normal control group.

Table S4. Effects on visceral fat, including mesenteric fat, subcutaneous fat, peritoneal fat and epididymal fat weights of rats fed with normal diet (NC), a high-fat diet (HFD) or HFD accompanied with treatment ECE at different doses of 125 mg (T1), 250 mg (T2), and 500 mg (T3) per kg B.W.

	Subcutaneous wt. (gm/rat)	Mesenteric wt. (gm/rat)	Peritoneal wt. (gm/rat)	Epididymal wt. (gm/rat)
**NC**	4.25 ± 2.96**	3.75 ± 1.78***	4.13 ± 1.64***	3.42 ± 1.34***
**HFD**	14.9 ± 8.23^{++}	8.4 ± 2.92	9.14 ± 2.33^{+++}	7.46 ± 2.00^{+++}
**HCA (250 mg/kg)**	11.06 ± 6.54	5.59 ± 1.14*	7.25 ± 1.81^{++}	6.12 ± 1.76^{++}
**EC (125 mg/kg)**	8.53 ± 3.71	5.84 ± 2.44	7.50 ± 1.54^{++}	5.63 ± 1.16^{+}
**EC (250 mg/kg)**	7.17 ± 2.42*	5.11 ± 2.22*	7.32 ± 1.22^{++}	4.98 ± 1.19*
**EC (500 mg/kg)**	6.49 ± 1.84*	4.62 ± 0.90**	5.84 ± 1.57**	4.75 ± 0.97**

Data are presented as means ± SEM (n = 8 for each group). * $p < 0.05$, ** $p < 0.01$, *** $p < 0.001$ vs. HFD group, + $p < 0.05$, ++ $p < 0.01$, and +++ $p < 0.001$ vs. normal control group.

Table S5. Effects on lipid profile of rats fed with normal diet; and high-fat diet with or without treatment of different doses of ECE (125, 250, and 500 mg/kg b.w) for 8 weeks.

Parameter	TC (mg/dL)	HDL (mg/dL)	LDL (mg/dL)	AI	Free-fatty acid (μM)	TG (mg/mL)
**NC**	121.53 ± 4.38***	102.87 ± 3.4***	18.67 ± 4.27***	120.53 ± 3.44***	0.26 ± 0.002***	282.86 ± 44.38***
**HFD**	193.54 ± 2.94+++	77.05 ± 5.78+++	116.49 ± 6.0+++	192.54 ± 3.81+++	0.79 ± 0.07+++	682.79 ± 14.59+++
**HCA (250 mg/kg)**	121.74 ± 4.37***	100.48 ± 7.1**	21.27 ± 5.14***	120.74 ± 1.34***	0.22 ± 0.01***	304.60 ± 86.25***
**EC (125 mg/kg)**	153.9 ± 3.7*** +++	87.66 ± 5.59+++	66.21 ± 3.5*** +++	152.9 ± 4.4***+++	0.52 ± 0.03	500.60 ± 42.48
**EC (250 mg/kg)**	125.51 ± 7.76***	91.76 ± 6.95*	33.74 ± 4.50***	124.51 ± 5.15***	0.42 ± 0.14*	331.28 ± 9.93***
**EC (500 mg/kg)**	119.82 ± 10.7***	97.75 ± 6.60**	22.07 ± 5.82***	118.82 ± 4.11***	0.37 ± 0.03**	226.37 ± 51.76***

Data are presented as means ± SEM (n = 8 for each group). * $p < 0.05$, ** $p < 0.01$, *** $p < 0.001$ vs. HFD group, + $p < 0.05$, ++ $p < 0.01$, and +++ $p < 0.001$ vs normal control group.

Table S6. Effects of *Ecklonia cava* ethanol (70%) extract (ECE) on plasma biomarkers as liver function enzymes in rats fed with normal diet; and high-fat diet with or without treatment of different doses of ECE (125, 250, and 500 mg/kg b.w) for 8 weeks.

Parameter	AST (U/L)	ALP (U/L)	ALT (U/L)	GGT (U/L)
NC	8.78 ± 0.96***	2.25 ± 0.35***	1.31 ± 0.18***	8.04 ± 1.25
HFD	17.62 ± 0.83+++	7.92 ± 0.93+++	7.79 ± 0.98+++	15.92 ± 6.43
HCA (250 mg/kg)	11.84 ± 1.48**	2.97 ± 0.18***	1.84 ± 0.53***	7.23 ± 0.57
EC (125 mg/kg)	13.78 ± 1.37+	4.49 ± 0.41+***	3.37 ± 0.46***	8.10 ± 7.78
EC (250 mg/kg)	13.09 ± 0.29*	4.26 ± 0.09+***	2.65 ± 0.27***	8.31 ± 5.8
EC (500 mg/kg)	10.87 ± 0.02**	2.61 ± 0.37***	1.68 ± 0.29***	8.50 ± 5.17

Data are presented as means ± SEM (n = 8 for each group). * $p < 0.05$, ** $p < 0.01$, *** $p < 0.001$ vs. HFD group, + $p < 0.05$, ++ $p < 0.01$ and +++ < 0.001 vs. normal control group.

Table S7. Comparison of different published studies related to anti-obesity effects of *Ecklonia cava* and current study.

Animal	Body + orgens + fat weight	Plasma biomarkers	Liver biomarkers	Glucose, Insuline	Histology Liver, Adipoe tissue	Gene expression	3T3-L1	Antioxident DPPH, ABTS assay	Ref
**Sprague–Dawley rats**	Yes	TC,TG,FFA, HDL, LDL, leptin, ghrelin, GIP	ALT, AST, GGT, AST	Yes	Yes	Yes	Yes	Both DPPH and ABTS	Our Manuscript
**C57BL/6N mice**	Yes	TC,HDL, leptin, GOT, GPT,	liver- TG	Yes	-	Yes	-	-	[16]
**C57BL/6NTacSam mice**	Yes	HDL, LDL, liptin	Liver- TG	Glucose	-	Yes	-	-	[17]
**C57BL/6N mice**	Yes	IL-6, TNF-a, IL-10	-	-	Adipos tissue	-	-	-	[18]
**Male C57BL/6 mice**	Yes	TG, TC, HDL, GOT,GPT	-	Glucose	Liver	Yes	-	-	[19]
**C57BL/6 mice**	Yes	TG, TC,HDL,LDL	ALT,AST	Glucose	-	Yes	-	-	[20]

Data presented in table is only for comparison prospective.

References

1. Laxmi, M.V.; Ravi, G.; Nath, A.R. Synthesis, Characterization of substituted 4-(bromomethyl)-N-(4-ethyl-6-methylpyrimidin-2-yl) benzamide Derivatives and Evaluation of their Anti-microbial activity.
2. Roh, C.; Jung, U.; Jo, S.-K. Screening of anti-obesity agent from herbal mixtures. *Molecules* **2012**, *17*, 3630-3638.
3. Shim, S.-L.; Hwang, I.-M.; Ryu, K.-Y.; Jung, M.-S.; Seo, H.-y.; Kim, H.-Y.; Song, H.-P.; Kim, J.-H.; Lee, J.-W.; Byun, M.-W. Effect of γ-irradiation on the volatile compounds of medicinal herb, Paeoniae Radix. *Radiation Physics and Chemistry* **2009**, *78*, 665-669.
4. Hifnawy, M.S.; Issaa, M.Y.; El-Seedi, H.; Mahrous, A.M.; Ashour, R. Phytochemical study, nutritional evaluation and in vitro antiobesity potential of fruits pericarp and seeds of Livistona carinensis and Thrinax parviflora. *Jordan Journal of Biological Sciences* **2021**, *14*.
5. Karabay-Yavasoglu, N.U.; Sukatar, A.; Ozdemir, G.; Horzum, Z. Antimicrobial activity of volatile components and various extracts of the red alga Jania rubens. *Phytotherapy Research: An International Journal Devoted to Pharmacological and Toxicological Evaluation of Natural Product Derivatives* **2007**, *21*, 153-156.

6. Roussis, V.; Tsoukatou, M.; Chinou, I.B.; Ortiz, A. Composition and Antibacterial Activity of the Essential Oils of Helichrysum rupestre and H. ambiguum Growing in the Balearic Islands1 (Part III). *Planta medica* **1998**, *64*, 675-676.
7. Fujita, K.i.; Chavasiri, W.; Kubo, I. Anti-Salmonella Activity of Volatile Compounds of Vietnam Coriander. *Phytotherapy Research* **2015**, *29*, 1081-1087.
8. Cruz, L.G.-d.l.; Caballero-Caballero, S.; Zamudio, S.; Duarte-Lisci, G.; Navarrete, A. Essential Oil Composition of Aerial Parts of Hypericum silenoides Juss. and Hypericum philonotis Cham. & Schlecht. Growing in Central Mexico §. *Journal of Essential Oil Bearing Plants* **2013**, *16*, 456-460.
9. Michel, P.; Granica, S.; Rosińska, K.; Rojek, J.; Poraj, Ł.; Olszewska, M.A. Biological and chemical insight into Gaultheria procumbens fruits: a rich source of anti-inflammatory and antioxidant salicylate glycosides and procyanidins for food and functional application. *Food & Function* **2020**, *11*, 7532-7544.
10. Salim, S.A. IN VITRO INDUCTION OF CALLUS FROM DIFFERENT EXPLANTS OFTERMINALIA ARJUNA (ROXB.) WIGHT AND ARN. AND DETECTION OF ITS ACTIVE SECONDARY METABOLITES USING GC-MS ANALYSIS. *Plant Archives* **2018**, *18*, 2519-2527.
11. Fadahunsi, O.; Adegbola, P.; Akintola, O.A.; Ajilore, B.S. The role of poly-herbal extract in sodium chloride-induced oxidative stress and hyperlipidemia in male Wistar rats. **2021**.
12. Swindell, E.P.; Hankins, P.L.; Chen, H.; Miodragović, Đ.U.; O'Halloran, T.V. Anticancer activity of small-molecule and nanoparticulate arsenic (III) complexes. *Inorganic chemistry* **2013**, *52*, 12292-12304.
13. Li, W.; Wang, P.J.; Shigematsu, M.; Lu, Z.G. Chemical composition and antimicrobial activity of essential oil from Amomum tsao-ko cultivated in Yunnan area. In Proceedings of the Advanced Materials Research, 2011; pp. 910-914.
14. Zhao, F.; Wang, P.; Lucardi, R.D.; Su, Z.; Li, S. Natural sources and bioactivities of 2, 4-di-tert-butylphenol and its analogs. *Toxins* **2020**, *12*, 35.
15. Varsha, K.K.; Devendra, L.; Shilpa, G.; Priya, S.; Pandey, A.; Nampoothiri, K.M. 2, 4-Di-tert-butyl phenol as the antifungal, antioxidant bioactive purified from a newly isolated Lactococcus sp. *International journal of food microbiology* **2015**, *211*, 44-50.
16. Kim, I.H.; Choi, J.W.; Lee, M.K.; Kwon, C.J.; Nam, T.J. Anti-obesity effects of pectinase and cellulase enzyme-treated Ecklonia cava extract in high-fat diet-fed C57BL/6N mice. *International journal of molecular medicine* **2018**, *41*, 924-934.
17. Kim, S.-Y.; Yun, I.-J.; Kwon, C.-J.; Choi, J.-W.; Kim, Y.-M.; Kang, M.-H.; Lee, M.-K.; Nam, T.-J. The effects of anti-obesity on enzyme-treated Ecklonia cava extracts. *Korean Journal of Fisheries and Aquatic Sciences* **2014**, *47*, 363-369.
18. Son, M.; Oh, S.; Choi, J.; Jang, J.T.; Choi, C.H.; Park, K.Y.; Son, K.H.; Byun, K. The phlorotannin-rich fraction of Ecklonia cava extract attenuated the expressions of the markers related with Inflammation and leptin resistance in adipose tissue. *International journal of endocrinology* **2020**, *2020*.
19. Eo, H.; Jeon, Y.-j.; Lee, M.; Lim, Y. Brown Alga Ecklonia cava polyphenol extract ameliorates hepatic lipogenesis, oxidative stress, and inflammation by activation of AMPK and SIRT1 in high-fat diet-induced obese mice. *Journal of agricultural and food chemistry* **2015**, *63*, 349-359.
20. Park, E.Y.; Kim, E.H.; Kim, M.H.; Seo, Y.W.; Lee, J.I.; Jun, H.S. Polyphenol-rich fraction of brown alga Ecklonia cava collected from Gijang, Korea, reduces obesity and glucose levels in high-fat diet-induced obese mice. *Evidence-based complementary and alternative medicine* **2012**, *2012*.
